# Polyclonal but not monoclonal circulating memory CD4^+^ T cells attenuate the severity of *Staphylococcus aureus* bacteremia

**DOI:** 10.3389/fimmu.2024.1417220

**Published:** 2024-05-29

**Authors:** Jessica Braverman, Ian R. Monk, Heran Zhang, Timothy P. Stinear, Linda M. Wakim

**Affiliations:** Department of Microbiology and Immunology, The University of Melbourne, at the Peter Doherty Institute for Infection and Immunity, Melbourne, VIC, Australia

**Keywords:** *Staphylococcus aureus* bacteremia, memory CD4^+^ T lymphocytes, polyclonal T cells, tissue-resident CD4^+^ T cells, circulating memory T-cell subsets

## Abstract

*Staphylococcus aureus* bacteremia causes significant morbidity and mortality. Treatment of staphylococcal infections is hindered by widespread antibiotic resistance, and attempts to develop an *S. aureus* vaccine have failed. Improved *S. aureus* treatment and infection prevention options require a deeper understanding of the correlates of protective immunity. CD4^+^ T cells have been identified as key orchestrators in the defense against *S. aureus*, but uncertainties persist regarding the subset, polarity, and breadth of the memory CD4^+^ T-cell pool required for protection. Here, using a mouse model of systemic *S. aureus* infection, we discovered that the breadth of bacterium-specific memory CD4^+^ T-cell pool is a critical factor for protective immunity against invasive *S. aureus* infections. Seeding mice with a monoclonal bacterium-specific circulating memory CD4^+^ T-cell population failed to protect against systemic *S. aureus* infection; however, the introduction of a polyclonal and polyfunctional memory CD4^+^ T-cell pool significantly reduced the bacterial burden. Our findings support the development of a multi-epitope T-cell-based *S. aureus* vaccine, as a strategy to mitigate the severity of *S. aureus* bacteremia.

## Introduction

*Staphylococcus aureus* is a Gram-positive bacterium and a common member of the human microbiota, occupying the skin and persistently colonizing the nares of approximately 30% of the human population (1). *Staphylococcus aureus* is also an opportunistic pathogen, capitalizing on breaches in mucosal barriers or cuts in the skin to gain access to deeper tissues. Here, the bacterium can cause life-threatening disease. *Staphylococcus aureus* bloodstream infections have high mortality rates (20%), frequent recurrences (5%–10%), and long-term sequelae in 34% of survivors (2–4). The management of invasive staphylococcal infections has become increasingly challenging due to the rise of multidrug-resistant strains (5, 6). While vaccination has proven effective at reducing the burden of disease against other problematic bacterial pathogens (7, 8), all *S. aureus* vaccine candidates thus far have failed in clinical trials (9). An effective vaccine against *S. aureus* would save many lives and potentially mitigate the rise of antimicrobial resistance.

Through their ability to boost phagocytic cell recruitment and their effector functions, CD4^+^ T cells play an important protective role against *S. aureus* infections (10–12). In general, the protective abilities of CD4^+^ T cells are influenced by the magnitude, breadth, location, polarity, and polyfunctionality of the T-cell pool (13). To develop a CD4^+^ T-cell vaccine that can elicit robust and durable protection against S. *aureus*, it is first necessary to define the characteristics of the CD4^+^ T-cell response required for *S. aureus* immunity. We have previously reported that bacterium-specific lung CD4^+^ Th1 polarized tissue-resident memory T cells (Trm) are crucial for the protection against *S. aureus* pneumonia (14). In the present study, we explored the type of bacterium-specific CD4^+^ T-cell memory required for protective immunity against *S. aureus* bacteremia. We show that, while monoclonal bacterium-specific circulating effector or memory CD4^+^ T cells failed to provide protection against systemic *S. aureus* infection, the introduction of a polyclonal and polyfunctional circulating memory CD4^+^ T-cell pool directed against three model T-cell antigens expressed by *S. aureus* significantly reduced the bacterial burden. Our results endorse the development of a multi-epitope T-cell-based *S. aureus* vaccine to reduce the severity of *S. aureus* bloodstream infections.

## Methods

### Ethics statement

All animal experiments were conducted in accordance with the Institutional Animal Care and Use Committee guidelines of the University of Melbourne and were approved by the University of Melbourne AEC (AEC 2015181; 27205).

### Mice

Female (6–14-week-old) C57BL/6 (CD45.2), RAG2^−/−^, μMT, gDT-2.CD45.1 (Vα3.2/Vβ2) (15), SMARTA.CD90.1 (Vα2.3/Vβ8) (16), and OT-2.CD45.1 (Vα2/Vβ5) transgenic mice were bred in-house and housed in specific pathogen-free conditions in the Biological Research Facility (BRF) at the Doherty Institute for Infection and Immunity, The University of Melbourne. All mouse strains used were shown to be *S. aureus* negative.

### Preparation of *Staphylococcus aureus* for inoculation and enumeration of bacterial loads

The streptomycin-resistant *S. aureus* strain JKD6159 and its recombinant derivatives were grown as previously described (17). Mice were inoculated intravenously with 10^6^ CFU of *S. aureus* in 200 μl of PBS. To evaluate *S. aureus* bacterial loads, tissue was harvested into 1 ml of PBS and homogenized, and serial dilutions of organ homogenates were made in PBS. Samples were plated onto brain heart infusion (BHI) agar plates containing 100 μg/ml of streptomycin and incubated overnight at 37°C.

### LCMV infections

Mice were infected intraperitoneally with 2 × 10^5^ PFU of Lymphocytic Choriomeningitis virus (LCMV) in 200 μl of PBS.

### Immunization of mice with heat-killed *Staphylococcus aureus*

Heat-killed *S. aureus* was generated as previously described (18). In brief, bacteria were grown in BHI broth at 37°C overnight with shaking (200 rpm), washed once in sterile PBS, and heat-killed in a 60°C heat block for 1 h. Mice were immunized twice by intraperitoneal injection of 10^8^ CFU of heat-killed *S. aureus* and 1 μg of the adjuvant lipopolysaccharide to boost inflammation, in 200 μl of PBS on days 0 and 7.

### Construction of *Staphylococcus aureus* strains (JKD-gp61 and JKD6159–3XEPI)

The gp61 (GP_61–80_) or OT-II (OVA_323–339_) epitopes were cloned into the pIME85(P*spa*-SD) plasmid as described previously (14). Briefly, the plasmid and PCR products (gp61: IM860/IM1694; OT-II: IM860/IM859) amplified from Newman genomic DNA were digested with NcoI/SacI, ligated, and transformed into DC10B-R with selection on L-agar containing 200 µg/ml of erythromycin. The pIME85(P*spa-*gp61-SD) plasmid was co-transformed (with pUCɸ85) into JKD6159Δ*hsdR*Δ*hsdR* ^SCC^, with the integrated plasmid transduced with ɸ11 into JKD6159^STR^ yielding JKD6159-gp61.

The JKD6159–3X^EPI^ strain was constructed by allelic exchange to introduce the OT-II and gD epitopes into JKD6159-gp61. A neutral intergenic site (between the convergent *icaC* and *lipA1*) proximal (~40 kb) to the origin of replication was identified in the JKD6159 chromosome. The region flanking the intergenic site was amplified by SOE-PCR (IM1862/IM1863 and IM1864/IM1865), introducing a unique NotI site and cloned into pIMAY-Z, yielding pIMAY-Z(*icaC*/*lipA1*). To assemble the epitopes, the following manipulations were conducted. The P*spa*-OTII-SD region was amplified from pIME85(P*spa*-OTII-SD) with primers (IM1826/IM1827) and cloned into pIME85 with BamHI/XbaI, yielding pIME85(OTII). The P*spa-*gD-SD was amplified from pIME85(P*spa*-gD-SD) (14) with primers (IM1822/IM1823) and cloned into pIME85(OTII) with KpnI/XhoI, yielding pIME85(gD/OTII). The gD/OTII cloned insert was amplified from pIME85(gD/OTII) with primers IM1866/IM1867, SLiCE cloned into pIMAY-Z(*icaC*/*lipA1*) was digested with NotI, and the backbone was amplified (IM1868/IM1869), yielding pIMAY-Z(*icaC*/gD+OTII/*lipA1*). The plasmid was directly transformed into JKD6159^STR^ (plasmid extracted from *Escherichia coli* IM93B), and allelic exchange was conducted as described by Monk and Stinear (19). All primer sequences are described in **Supplementary Table 1**.

### Assessment of cytokines via cytometric bead array

Cytokine/chemokine concentrations in serum were measured using a LegendPlex mouse antiviral response cytometric bead array (BioLegend San Diego, CA, USA) kit following the manufacturer’s instructions.

### Purification and adoptive transfer of naive murine T cells

CD4^+^ T cells isolated gDT-2.CD45.1, SMARTA.CD90.1, or OT-2.CD45.1 were purified from single-cell suspensions prepared from the lymph nodes (LNs) and spleen via negative selection using previously described protocols (20). Mice received 5 × 10^4^ or 1 × 10^6^ carboxyfluorescein succinimidyl ester (CFSE)-labeled naive transgenic T cells intravenously in a volume of 200 μl.

### Generation of mice with T-cell memory

gDT-2.CD45.1, SMARTA.Thy1.1, and OT-2.CD45.1 CD4^+^ T cells were activated *in vitro* with 10^−6^ M of gD_315–327_ (IPPNWHIPSIQDA) peptide or 10^–6^ M of GP_61–80_ (GLKGPDIYKGVYQFKSVEFD) or 10^−6^ M of OVA_323–339_ (ISQAVHAAHAEINEAGR) peptide-pulsed splenocytes, respectively, as previously described (21). Mice were injected intravenously with 5 × 10^6^ *in-vitro*-activated effectors and rested for at least 20 days to establish circulating memory T cells.

### Flow cytometry and intracellular cytokine staining

Single-cell suspensions were prepared from the spleen and LN by mechanical disruption. Mice were perfused with PBS before the harvest of the lung, nose, liver, and kidney tissue, which were then enzymatically digested for 1 h at 37°C in 3 ml of collagenase type 3 [3 mg/ml in RPMI 1640 medium supplemented with 2% fetal bovine serum (FBS)]. Lymphocytes were enriched from the liver and kidney using a Percoll gradient. Cells were incubated with the appropriate cocktail of monoclonal antibodies (mAbs) for 30 min on ice. For intracellular cytokine analysis, single-cell suspensions of the lung and spleen were stimulated with either the 1 μM of cognate peptide [OVA_323–339_ (ISQAVHAAHAEINEAGR); gp_61–80_ (GLKGPDIYKGVYQFKSVEFD); gD_315–327_ (IPPNWHIPSIQDA)] for 5 h at 37°C/10% CO_2_ in the presence of GolgiPlug (BD Biosciences) in complete RPMI [10% FBS, 2 mM of glutamine, 50 mM of 2-β mercaptoethanol (2-ME), penicillin (100 U/ml), and streptomycin (100 μg/ml)]. Cells were then surface-stained for 30 min on ice with the appropriate mixture of mAbs and then intracellularly stained using a Foxp3 fix/perm kit (Thermo Fisher Scientific) according to the manufacturer’s protocol. The conjugated mAbs obtained from BD Pharmingen (Franklin Lakes, NJ, USA), BioLegend (San Diego, CA, USA), or eBioscience (San Diego, CA, USA) include mouse: anti-CD8 (53–6.7), anti-CD45.1 (A20), anti-CD44 (1M7), anti-CD103 (2E7), anti-CD69 (H1.2F3), anti-IFNγ (XMG1.2), anti-TNFα (MP6-XT22), anti-CD62L (MEL-14), anti-IL-17 (TC11), anti-CD4 (GK1.5), and anti-90.1 (OX.7). Samples were acquired using a Becton Dickinson LSRFortessa flow cytometer, and data were analyzed using the FlowJo software package (Tree Star Inc., Ashland, OR, USA).

### *In-vitro* antigen presentation assay

Dendritic cells were enriched from enzymatically digested spleens collected from B6 mice as previously described (20). Dendritic cells (DCs; 5 × 10^4^) were cultured 1:1 with CFSE-labeled CD4^+^ T cells (enriched from either naive SMARTA.CD90.1, OT-2.CD45.1, or gDT-2.CD45.1 mice) for 3 days either alone or with titrated doses of heat-killed *S. aureus* in complete RPMI [10% FBS, 2 mM of glutamine, 50 mM of 2-β mercaptoethanol (2-ME), penicillin (100 U/ml)].

### Immunohistochemistry

Perfused kidney tissue was fixed in 4% paraformaldehyde for 3 h on ice and then placed in titrated concentrations of sucrose at 4°C (5% sucrose overnight, 15% sucrose for 3 h, 30% sucrose for 3 h) and then embedded in OCT. Frozen sections (14 μm) were cut using a cryostat. Tissue sections were fixed with acetone and blocked in serum-free protein block (DAKO, Santa Clara, CA, USA). Sections were stained with a polyclonal rabbit anti*-S. aureus* antibody (Thermo Fisher, Cat#PA1–7246, Waltham, MA, USA), anti-Ly6g (1A8), and DAPI. The coverslips were mounted in Prolong Gold Antifade medium. Slides were imaged using a Zeiss LSM700 microscope and images were analyzed using ImageJ (version 2).

### Statistical analysis

Comparison between the two study groups was statistically evaluated by unpaired two-tailed *t*-test or Mann–Whitney test. Comparison between more than two groups (single factor) was evaluated using one-way analysis of variance (ANOVA) with Tukey’s or Dunnett’s multiple comparison. Two-way ANOVA with Sidak’s multiple comparison on log10-transformed values was used to evaluate more than two groups at different time points. In all tests, statistical significance was quantified as *P <0.5, **P <0.01, ***P <0.001, and ****P <0.0001. Statistical analysis was performed using GraphPad Prism 8 software.

## Results and discussion

### Development of a mouse model of *Staphylococcus aureus* bacteremia

**Figure 1:**
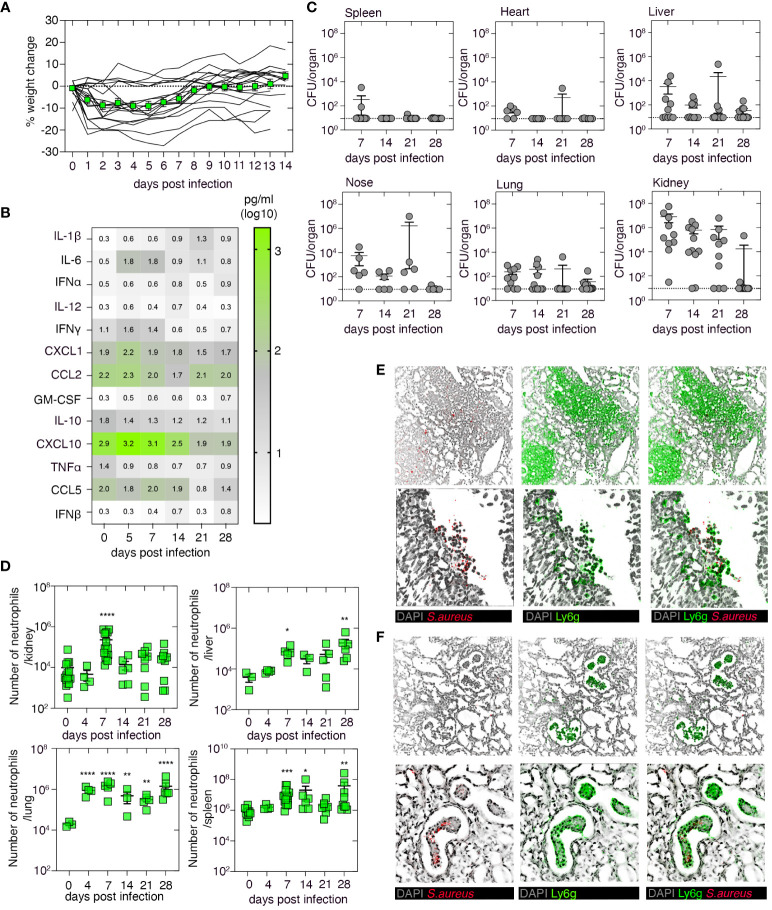
Characterization of the systemic *Staphylococcus aureus* infection model. C57BL/6 mice were infected i.v. with 10^6^ CFU of the *S. aureus* strain JKD6159. **(A)** The graph depicts the percentage weight change measured over the course of the experiment. Lines represent individual mice (*n* = 18); the green line represents mean ± SEM. **(B)** Heatmap shows the level of a panel of cytokines/chemokines in the serum at the indicated days after infection, measured by cytometric bead array (*n* = 4–20 mice/time point). **(C)** Bacterial loads in the spleen, heart, liver, nasal tissue, lung, and kidney were measured at the indicated days after infection. Symbols represent individual mice, and the bars represent the mean + SEM (*n* = 10–12/time point). **(D)** C57BL/6 mice were infected i.v. with 10^6^ CFU of JKD6159, and the number of neutrophils (Ly6g^+^CD11b^+^) in the kidney, liver, lung, and spleen at the indicated days after infection was measured. Symbols represent individual mice, and bars represent mean + SEM (*n* = 6–12 mice/time point; one-way ANOVA, Dunnett’s multiple comparison). **(E, F)** Microscopy of mouse kidney tissue on days **(E)** 7 and **(F)** 14 after i.v. infection with 10^6^ CFU of JKD6159, stained with anti-*S. aureus* (red), anti-Ly6g (green), and DAPI (gray); top panel ×20; bottom panel ×40 *P <0.5, **P <0.01, ***P <0.001, and ****P <0.0001.

We first established and characterized a systemic mouse model of *S. aureus* infection. To do this, C57BL/6 mice were infected intravenously (i.v.) with 10^6^ CFU of the *S. aureus* strain JKD6159. While weight loss following infection was highly variable, with animals losing ~10% of their body weight, all animals survived the infection (**Figure 1A**). The introduction of *S. aureus* into the bloodstream induced transient, systemic inflammation, and consistent with earlier reports (22), significantly elevated levels of IL-6 were detected in the serum both 5 and 7 days after infection (**Figure 1B**). Assessment of the bacterial load in the spleen, heart, liver, nasal tissue, lung, and kidney revealed the kidney as the major reservoir of infection, with high titers of *S. aureus* being detected in this organ in >70% of the animals for at least 21 days after infection (**Figure 1C**). While *S. aureus* was also present in the spleen, heart, liver, nasal tissue, and lung, bacterial loads within these tissues were generally low and variable (**Figure 1C**). Inflammation, as measured by an increase in the number of neutrophils, was evident in the lung, kidney, liver, and spleen, and while neutrophil numbers peaked in the kidney on day 7 p.i. and then returned to baseline levels, neutrophil numbers in all the other organs remained significantly elevated for at least 28 days after infection (**Figure 1D**). Histology of the kidneys on day 7 p.i. confirmed the presence of large swarms of neutrophils and *S. aureus* bacteria; however, by day 14 p.i., this inflammation had subsided and the development of renal abscesses, which is a characteristic feature of systemic staphylococcal infections, was evident (**Figures 1E, F**). Thus, bloodstream infection of C57BL/6 mice with JKD6159 resulted in the development of *S. aureus* bacteremia.

### CD4^+^ T cells are involved in the clearance of systemic *Staphylococcus aureus* infection

**Figure 2:**
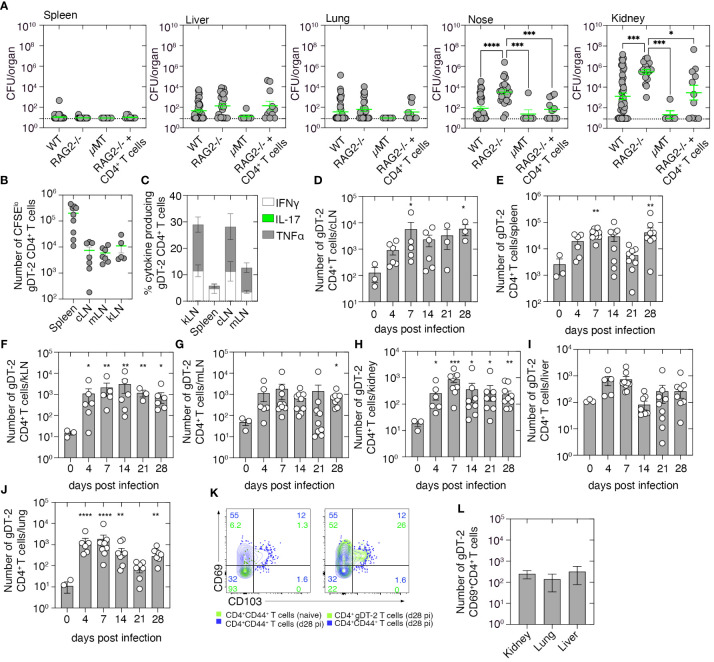
Tracking the CD4^+^ T-cell response to systemic *Staphylococcus aureus* infection. **(A)** C57BL/6 mice (WT), RAG2^−/−^ mice, μMT mice, or RAG2^−/−^ mice seeded with naive 5 × 10^6^ CD4^+^ T cells derived from C57BL/6 mice were infected i.v. with 10^6^ CFU of JKD6159, and bacterial loads in the spleen, liver, nasal tissue, lung, and kidney were measured 21 days after infection. Symbols represent individual mice, and the bars represent the mean ± SEM (*n* = 5–44; one-way ANOVA, Tukey’s multiple comparison). **(B)** C57BL/6 mice seeded with 1 × 10^6^ CFSE-labeled gDT-2.CD45.1^+^ CD4^+^ T cells were infected i.v. with 10^6^ CFU of JKD6159-gD, and 1–4 days later, the number of divided (CFSE^lo^) gDT-2.CD45.1^+^ CD4^+^ T cells was measured. **(B)** The absolute number of divided (CFSE^lo^) gDT-2 CD4^+^ T cells in the cLN, mLN, kLN, or spleen and **(C)** the proportion expressing TNFα, IFNγ, or IL-17 following a brief *in-vitro* stimulation in the spleen, kLN, cLN, and mLN on day 4 after infection. Symbols represent the mean ± SEM (*n* = 4–9 mice/time point; two-way ANOVA, Dunnett’s multiple comparison). **(D–L)** C57BL/6 mice seeded with naive 5 × 10^4^ gDT-2.CD45.1^+^ CD4^+^ T cells and infected i.v. with 10^6^ CFU of JKD6159-gD, and 0–28 days later, the number of gDT-2.CD45.1^+^ CD4^+^ T cells in the **(D)** cLN, **(E)** spleen, **(F)** kLN, **(G)** mLN, **(H)** kidney, **(I)** liver, and **(J)** lung was measured. Symbols represent individual mice with the bars representing the mean ± SEM (*n* = 6–10 mice/time point; one-way ANOVA, Dunnett’s multiple comparison). **(K)** Representative flow cytometry profiles showing the level of expression of CD69 and CD103 on gDT-2.CD45.1^+^ CD4^+^ T cells and endogenous memory CD4^+^CD44^+^ T cells in the kidney of mice infected i.v. 28 days earlier with 10^6^ CFU of JKD6159-gD. As a control, the expression of CD69 and CD103 on CD4^+^CD44^+^ T cells from uninfected mice is also depicted. **(L)** The absolute number of CD69^+^ gDT-2.CD45.1^+^ CD4^+^ T cells in the kidney, lung, and liver on day 28 after infection (*n* = 6–10 mice) *P <0.5, **P <0.01, ***P <0.001, and ****P <0.0001.

To examine whether adaptive immunity plays a role in the clearance of a systemic *S. aureus* infection, we i.v. infected RAG2^−/−^ mice, which lack both T and B cells, μMT mice which lack only B cells, or as a control, wild-type C57BL/6 mice, with 10^6^ CFU of *S. aureus* (JKD6159), and 21 days later, we measured bacterial loads in various tissues. While low levels of bacteria were present in the spleen, liver, and lung of both wild-type and immunocompromised mice (**Figure 2A**), there was an increased bacterial burden (~100-fold) in the nose and kidney of RAG2^−/−^ mice when compared to the control wild-type cohort (**Figure 2A**). This impairment in bacterial clearance in RAG2^−/−^ mice appeared linked to the absence of the T-cell compartment, as μMT mice infected with *S. aureus* eliminated the bacteria from the nasal passage and kidney at a rate like that observed in wild-type control animals (**Figure 2A**). To confirm this and to identify the T-cell subset responsible for *S. aureus* clearance from these tissues, we adoptively transferred into RAG2^−/−^ mice polyclonal CD4^+^ T cells purified from the spleens of naive C57BL/6 mice, and 1 day later, we infected these animals intravenously with 10^6^ CFU of *S. aureus* (JKD6159). Assessment of the bacterial burden on day 21 p.i. revealed that the transfer of polyclonal CD4^+^ T cells alone significantly lowered the bacterial load in both the nasal tissue and kidney of RAG2^−/−^ mice, confirming that CD4^+^ T-cell immunity is important for the clearance of systemic *S. aureus* infection (**Figure 2A**).

We next set out to track the *S. aureus*-specific CD4^+^ T-cell response generated following systemic infection. As endogenous *S. aureus* CD4^+^ T-cell epitopes are currently undefined, we used a previously described model which involved the use of a recombinant *S. aureus* strain that expresses a CD4^+^ T-cell epitope derived from herpes simplex virus (HSV) glycoprotein D (gD) and T-cell receptor (TCR) transgenic CD4^+^ gDT-2 cells which are specific for class II-restricted HSV gD (14). We first determined the site where CD4^+^ gDT-2 T-cell priming occurs following systemic JKD6159-gD infection. To this end, C57BL/6 recipient mice (CD45.2^+^) were injected with 1 × 10^6^ CFSE-labeled gDT-2.CD45.1^+^ cells 1 day prior to i.v. infection with 10^6^ CFU of JKD6159-gD. By day 4 p.i., sizable populations of divided gDT-2 CD4^+^ T cells (CFSE^lo^) were detected in the spleen, cervical lymph node (cLN), mediastinal LN (mLN), and kidney draining LN (kLN) (**Figure 2B**); moreover, these cells where largely IFNγ and TNFα producing Th1 (**Figure 2C**). Thus, CD4^+^ T-cell priming occurs systemically in the spleen, cLN, mLN, and kLN, following a bloodstream *S. aureus* infection.

Next, we monitored the expansion, infiltration, and persistence of gDT-2 CD4^+^ T cells into bacteria-infected tissue. Naive gDT-2.CD45.1 CD4^+^ T cells were transferred into C57BL/6 recipient mice (CD45.2^+^) prior to i.v. infection with 10^6^ CFU of JKD6159-gD, and the number of gDT-2 CD4^+^ T cells (Vα3.2^+^CD45.1^+^) in various tissue compartments, including the cLN, mLN, kLN, spleen, lung, liver, and kidney, was measured. Bloodstream infection with JKD6159-gD resulted in gDT-2 CD4^+^ T-cell expansion, and the number of gDT-2.CD4^+^ T cells in all tissues profiled peaked on day 7 after infection and remained significantly elevated in all tissues, except the liver, until day 28 post-challenge (**Figures 2D–J**). Assessment of the expression of the Trm markers on gDT-2 CD4^+^ T cells in the kidney on day 28 post-challenge revealed that >70% of gDT-2 CD4^+^ T cells within this compartment were CD69^+^ (**Figure 2K**), which is indicative of tissue residency, and a similar number of CD69^+^ gDT-2^+^ CD4^+^ T cells were also detected in both the lung and liver (**Figure 2L**). These findings show that systemic infection with *S. aureus* results in the activation and expansion of bacterium-specific CD4^+^ T cells which disseminate and persist within infected internal organs.

### Monoclonal *Staphylococcus aureus*-specific effector and memory CD4^+^ T cells fail to prevent bacteremia

**Figure 3:**
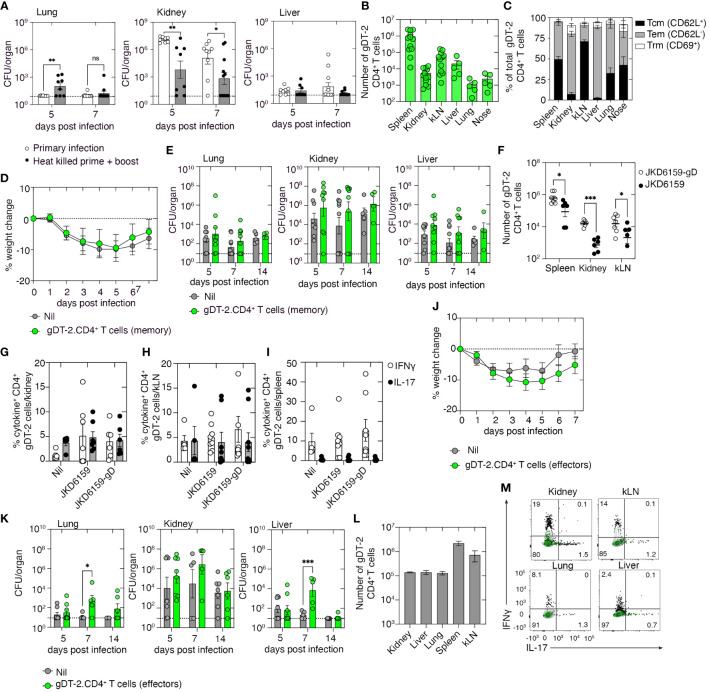
Memory and effector gDT-2.CD4^+^ T cells fail to protect against systemic *Staphylococcus aureus* infection. **(A)** C57BL/6 mice (primary) or C57BL/6 mice prime and boosted with heat-killed *S. aureus* + adjuvant 14 days prior were infected i.v. with 10^6^ CFU JKD6159. Bacterial loads in the lung, kidney, and liver at the indicated time points were measured. Symbols represent individual mice, and the bars represent the mean ± SEM (*n* = 8–9 mice/time point, two-way ANOVA, Sidak’s multiple comparison). **(B, C)** C57BL/6 mice seeded with 5 × 10^6^ *in-vitro*-activated effector gDT-2.CD45.1^+^ CD4^+^ T cells and rested for 20 days. **(B)** The number of gDT-2.CD45.1^+^ CD4^+^ T cells and **(C)** the proportion expressing Tcm (CD62L^+^), Tem (CD62L^−^), and Trm (CD69^+^) markers in various tissues were measured. Symbols represent individual mice, and the bars represent the mean ± SEM (*n* = 5–12 mice). **(D, E)** C57BL6 mice and C57BL/6 mice with memory gDT-2.CD45.1^+^ CD4^+^ T cells were infected i.v. with 10^6^ CFU JKD6159-gD. **(D)** Weight loss and **(E)** bacterial load in the kidney, nose, lung, spleen, and liver were measured on days 5, 7, and 14 after infection. Symbols represent individual mice, and the bars represent the mean ± SEM (*n* = 9–10 mice/time point). **(F–I)** C57BL/6 mice with memory gDT-2.CD45.1^+^ CD4^+^ T cells were infected i.v. with 10^6^ CFU of JKD6159-gD or JKD6159, and 5 days later, the spleen, kidney, and kLN were collected and the **(F)** number gDT-2 CD4^+^ T cells and the proportion of gDT-2 CD4^+^ T cells in the **(G)** kidney, **(H)** kLN, and **(I)** spleen producing IFNγ and IL-17 following a brief *in-vitro* stimulation were measured. Symbols represent individual mice, and the bars represent the mean ± SEM (*n* = 5–8 mice/time point, two-way ANOVA, Tukey’s multiple comparison). **(J–M)** C57BL/6 mice and C57BL/6 mice seeded with effector gDT-2.CD45.1^+^ CD4^+^ T cells were infected i.v. with 10^6^ CFU JKD6159-gD. **(J)** The graph depicts weight loss over the course of the experiment. **(K)** Bacterial loads in the kidney, lung, and liver were measured at the indicated time points. Symbols represent individual mice, and the bars represent the mean ± SEM (*n* = 5–8 mice/time point, two-way ANOVA, Sidak’s multiple comparison). **(L)** The number of effector gDT-2 CD4^+^ T cells in the kidney, liver, lung, spleen, and kLN on day 5 after infection and **(M)** representative flow cytometry profiles depicting the proportion of gDT-2 CD4^+^ T cells producing IFNγ and IL-17 following a brief *in-vitro* stimulation was measured. Green contour plots represent unstimulated controls *P <0.5, **P <0.01, ***P <0.001, and ****P <0.0001. ns, non significant.

We have shown that primary infection with *S. aureus* fails to result in the development of protective immunity against a secondary invasive *S. aureus* infection (**Supplementary Figure 1**). However, mice can build up immunity against systemic *S. aureus* infection following repeated exposures. To demonstrate this response, we primed and boosted mice with heat-killed *S. aureus* co-administered with the adjuvant lipopolysaccharide (LPS) and 2 weeks later challenged these animals, and as a control, a naive cohort, i.v. with 10^6^ CFU of JKD6159. Bacterial loads in the lung, kidney, and liver were measured 5 and 7 days post-challenge. On day 5 p.i., animals immunized with heat-killed *S. aureus* exhibited significantly lower bacterial loads in the kidney compared to the control cohort, a trend that was also evident on day 7 p.i. (**Figure 3A**). We then tested if protective immunity against invasive *S. aureus* bloodstream infection could be achieved with anti-*S. aureus* CD4^+^ T-cell immunity alone. To do this, animals with a sizable pool of circulating *S. aureus*-reactive CD4^+^ memory T cells were generated by injecting mice with 5 × 10^6^ *in-vitro*-activated effector gDT-2 CD4^+^ T cells and then resting these mice for 20 days; we have previously shown that this technique results in sizable populations of predominantly Tem and Tcm, but few Trm (**Figures 3B, C**). Animals with circulating memory gDT-2 CD4^+^ T cells, and as a control, a naive cohort, were challenged i.v. with 10^6^ CFU of JKD6159-gD. Weight loss, a readout of disease severity, and bacterial loads in the lung, kidney, and liver were measured on days 5, 7, and 14 p.i. Comparable weight loss (maximum ~10%) (**Figure 3D**) and similar bacterial loads in all the tissues at all the time points profiled (**Figure 3E**) were observed in both cohorts. Thus, circulating bacterium-specific memory CD4^+^ T cells alone are insufficient to prevent systemic *S. aureus* infection.

To understand why circulating memory CD4^+^ T cells did not confer protection against *S. aureus* bacteremia, we examined the recall response of these cells. Mice with circulating memory gDT-2 CD4^+^ T cells, generated as described above, were challenged i.v. with either JKD6159-gD or as a control, the parental strain, JKD6159. Quantitation of the number of gDT-2 CD4^+^ T cells in the spleen, kidney, and kLN on day 5 post-challenge revealed that compared to mice challenged with the parental strain, animals challenged with the gD-expressing strain of *S. aureus* contained elevated numbers of gDT-2 CD4^+^ T cells in all tissues profiled (**Figure 3F**). Assessment of the cytokine profile of these gDT-2 CD4^+^ T cells following a brief *in-vitro* stimulation with cognate peptide revealed equal proportions (~5%) of IL-17^+^ and IFNγ^+^ gDT-2 CD4^+^ T cells in the kidney and kLN across all cohorts (**Figures 3G, H**), while gDT-2 CD4^+^ T cells in the spleen of all cohorts were predominantly IFNγ producing Th1 cells (**Figure 3I**). These data show that despite bacterium-specific circulating memory CD4^+^ T cells being present within infected organs and displaying intact effector functions, these cells failed to prevent systemic *S. aureus* infection.

We next checked whether the presence of high numbers of bacteria-specific CD4^+^ effectors T cells early during the infection could attenuate the severity of *S. aureus* bacteremia. To do this, we seeded mice with *in-vitro*-activated effector gDT-2 CD4^+^ T cells and, on the same day, infected these animals, and as a control, a naive cohort, i.v. with 10^6^ CFU of JKD6159-gD, and as above, we monitored weight loss and bacterial loads as readouts of disease severity. Both mice seeded with effector gDT-2 CD4^+^ T cells and the control cohort lost similar amounts of weight following systemic *S. aureus* challenge (**Figure 3J**). Unexpectedly, while the bacterial burden in the kidney was equivalent across both cohorts at all time points, on day 7 p.i., mice seeded with bacterium-specific effector CD4^+^ T cells harbored 500–1,000-fold higher levels of *S. aureus* when compared to the control cohort, in both the lung and liver (**Figure 3K**). These data suggest that large numbers of bacterium-specific effector CD4^+^ T cells early during infection can exacerbate a systemic *S. aureus* infection. However, this effect was transient as bacterial loads on day 14 p.i. within these tissue compartments matched those in the control cohort (**Figure 3K**). We checked the size and polarity of this effector gDT-2 CD4^+^ T-cell pool following the *S. aureus* challenge and confirmed that by day 5 post-challenge large numbers of effector gDT-2 CD4^+^ T cells were present in the kidney, liver, lung, spleen, and kLN (10^5^–10^6^ cells) (**Figure 3L**), and these effector cells were predominantly producing Th1 cytokines (**Figure 3M**). Collectively, these data show that bacterium-specific circulating effector or memory CD4^+^ Th1 cells directed against a single bacterial epitope cannot prevent *S. aureus* bacteremia.

### Boosting bacterium-specific CD4^+^ Trm within internal organs enhances protection against systemic *Staphylococcus aureus* infection

**Figure 4:**
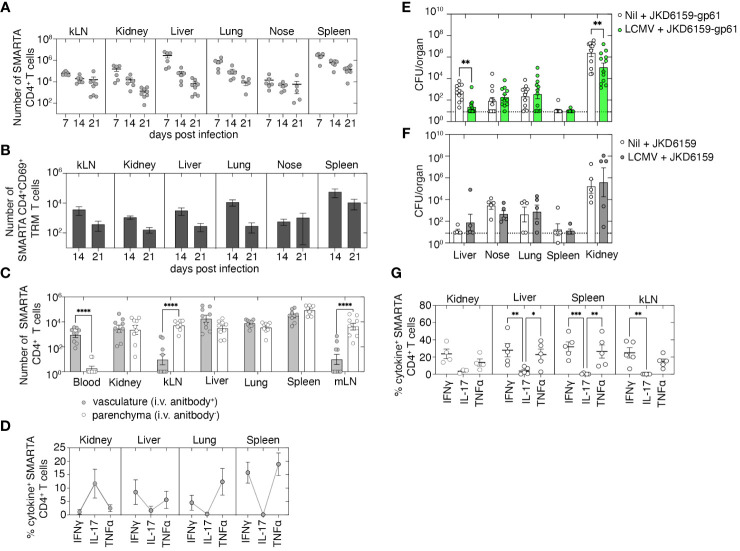
Boosting local and circulating *Staphylococcus aureus* reactive memory CD4^+^ T cells improves bacterial control following systemic infection. **(A–D)** C57BL/6 mice seeded with 5 × 10^4^ naive SMARTA.CD90.1^+^ CD4^+^ T cells and infected i.p. with 2 × 10^5^ PFU of LCMV (Armstrong) and on days 7, 14, and 21 after infection, just prior to the harvest, mice were injected i.v. with anti-CD4. The number of **(A)** total, **(B)** CD69^+^, and **(C)** vasculature and parenchyma localized SMARTA.CD90.1^+^ CD4^+^ T cells in the kLN, kidney, liver, lung, blood, nasal tissue, and spleen was measured. Symbols represent individual mice, and bars represent mean ± SEM (*n* = 8). **(D)** The proportion of SMARTA.CD90.1^+^ CD4^+^ T cells producing IFNγ, TNFα, and IL-17 following a brief *in-vitro* stimulation was measured. Symbols represent individual mice, and bars represent mean + SEM (*n* = 8). **(E–G)** C57BL/6 mice and C57BL/6 mice infected i.p. with 2 × 10^5^ PFU of LCMV (Armstrong) 21 days prior were infected i.v. with either **(E)** JKD6159-gp61 or **(F)** the parental strain JKD6159, and on day 7 p.i., the bacterial loads in the liver, nasal tissue, lung, spleen, and kidney were measured. Symbols represent individual mice, and the bars represent the mean ± SEM. The dotted line shows the limit of detection (*n* = 5–12 mice per group, two-way ANOVA, Sidak’s multiple comparison). **(G)** The proportion of SMARTA.CD90.1^+^ CD4^+^ T cells on day 7 after JKD6159-gp61 producing IFNγ, IL-17, and TNFα following a brief *in-vitro* stimulation. Symbols represent individual mice, and bars represent the mean ± SEM (*n* = 4–5 mice group, two-way ANOVA, Tukey’s multiple comparison) *P <0.5, **P <0.01, ***P <0.001, and ****P <0.0001.

We next examined whether boosting bacterium-specific CD4^+^ Trm within internal organs that represent the primary sites of bacterial replication following a systemic *S. aureus* infection would improve disease outcomes. Previously, it has been demonstrated that systemic LCMV infection results in the development of virus-specific circulating memory T cells as well as the deposition of Trm in several internal organs including the kidney, lung, and liver (23). We used this virus as a means to create circulating LCMV-specific CD4^+^ T cells, as well as Trm in the kidney, liver, and lung, and then, we tested the protective capacity of this immune profile against a systemic *S. aureus* infection by challenging these animals with *S. aureus* JKD6159-gp61, a strain we engineered to express the LCMV CD4^+^ T-cell epitope, gp_61–80_. We first confirmed, consistent with prior reports (23), that LCMV infection did boost virus-specific CD4^+^ Trm within the kidney, lung, and liver. C57BL/6 mice were seeded with naive CD4^+^ T cells that were purified from the SMARTA.CD90.1 CD4^+^ TCR transgenic mice that are specific for the class II-restricted LCMV-derived gp_61–80_ epitope. We then infected these animals i.p. with LCMV (Armstrong). Following LCMV infection, SMARTA.CD90.1 CD4^+^ T cells expanded, and the numbers peaked on day 7 after infection and contracted thereafter. However, sizable memory SMARTA.CD90.1^+^ CD4^+^ T cells could still be detected in the kLN, kidney, liver, lung, nasal tissue, and spleen on day 21 p.i. (**Figure 4A**). Notably, a significant proportion of these cells in the kidney, liver, and lung (45%, 17%, and 32%, respectively) were parenchyma-bound CD69^+^ Trm (**Figures 4B, C**). Assessment of the cytokine profile of SMARTA memory CD4^+^ T cells revealed that following a brief *in-vitro* stimulation, the majority of SMARTA CD4^+^ T cells in the liver, lung, and spleen were Th1-polarized IFNγ or TNFα producers, while in the kidney, these cells were largely IL-17 producers (**Figure 4D**). To assess whether this type of immunity could confer protection against a systemic *S. aureus* infection, naive C57BL/6 mice or C57BL/6 mice seeded with naive SMARTA.CD90.1 CD4^+^ T cells and infected i.p. with LCMV 21 days prior were i.v. infected with 10^6^ CFU of either JKD6159-gp61 or, as a control, the parental strain JKD6159. Bacterial loads in various internal organs after *S. aureus* challenge were measured on day 7 after infection. LCMV-primed mice challenged with JKD6159-gp61 harbored significantly fewer bacteria in the liver and kidney compared to the naive cohort that was challenged with this strain (**Figure 4E**). This improved clearance of bacteria from the kidney and liver was antigen-specific, for when LCMV-primed mice were challenged with the parental *S. aureus* strain, which lacks the gp61 epitope, no reduction in bacterial loads was observed (**Figure 4F**). Assessment of the cytokine profile of SMARTA CD4^+^ T cells on day 7 after JKD6159-gp61 infection revealed that the majority of SMARTA.CD90.1 CD4^+^ T cells within the kidney, liver, spleen, and kLN were producing IFNγ or TNFα, but not IL-17 (**Figure 4G**). These data show that boosting monoclonal bacteria-specific memory CD4^+^ Th1-polarized circulating and internal organ Trm can significantly reduce *S. aureus* bacteremia severity.

### Polyclonal bacteria-specific memory CD4^+^ T-cell response attenuates *Staphylococcus aureus* bacteremia

**Figure 5:**
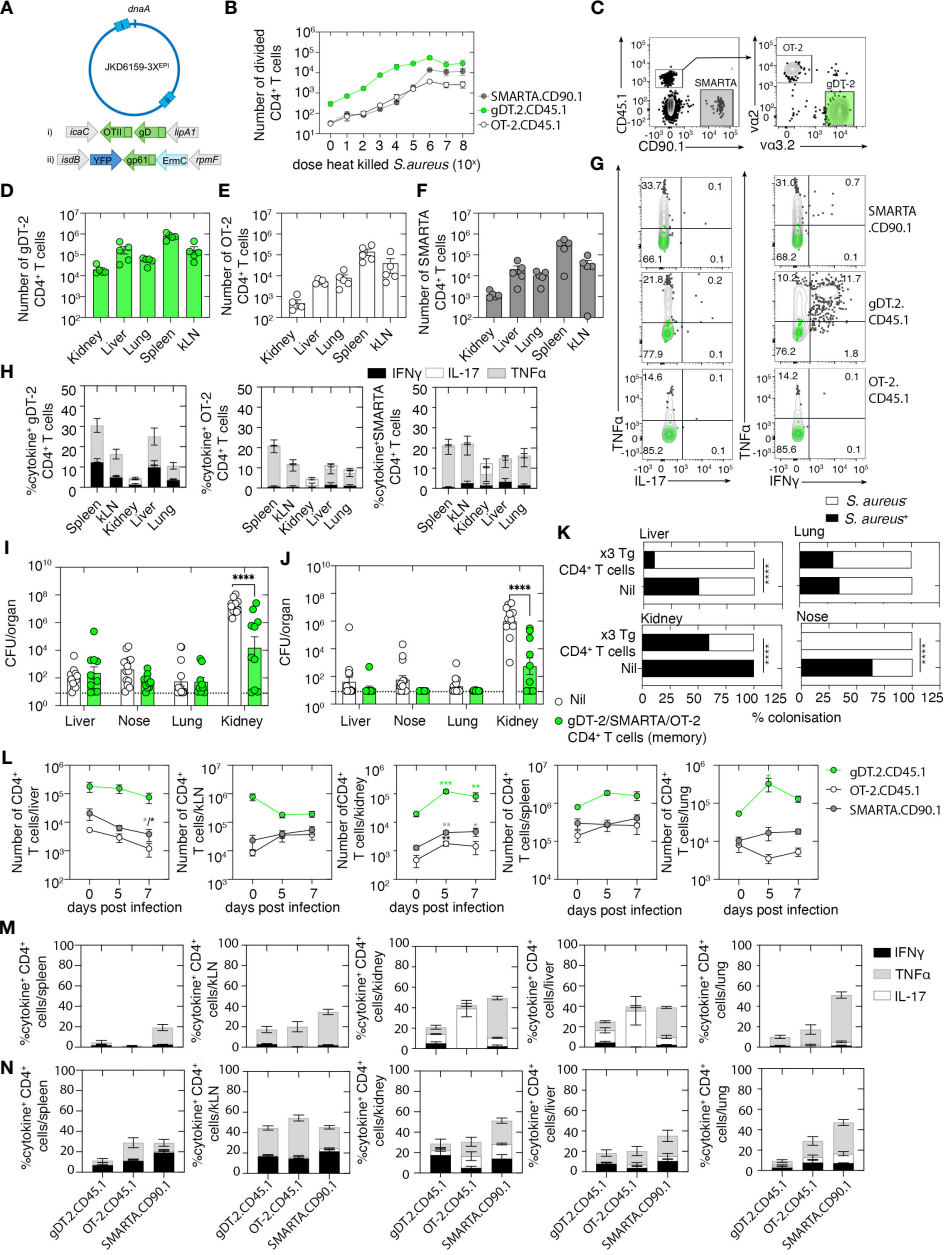
A polyclonal circulating memory CD4^+^ T-cell response directed against *Staphylococcus aureus* reduces the severity of bacteremia. **(A)** Schematic of the JKD6159–3X^EPI^ chromosome highlighting the sites of epitope integration: i) OTII/gD integrated by allelic exchange between the convergent *icaC*-*lipA1* genes and ii) gp61 integrated between the convergent *isdB-rpmF* genes on the phage integrase plasmid pIME85. All three epitopes are expressed from the upstream P*spa* promoter. **(B)** 5 × 10^4^ CFSE-labeled gDT-2 CD45.1 or SMARTA.CD90.1 or OT-2.CD45.1 CD4^+^ T cells and 5 × 10^4^ splenic dendritic cells were cultured with varying doses (10^0^–10^8^ CFU) of heat-killed JKD6159–3X^EPI^ and the proportion of divided CD4^+^ T cells was measured 60 h later. The graph depicts the absolute number of divided (CFSE^lo^) CD4^+^ T cells. Symbols represent the mean ± SEM. Data are representative of three independent experiments. **(C–G)** C57BL/6 mice were seeded with 5 × 10^6^ *in-vitro*-activated gDT-2.CD45.1, OT-2.CD45.1, and SMARTA.CD90.1 CD4^+^ T cells and were rested for 20 days. **(C)** Representative flow cytometry plots show the gating strategy to identify the three transgenic CD4^+^ T-cell populations. **(D–G)** The absolute number of **(D)** gDT-2.CD45.1, **(E)** OT-2.CD45.1, and **(F)** SMARTA.CD90.1 CD4^+^ T cells in the kidney, liver, lung, spleen, and kLN. Symbols represent individual mice, and bars represent the mean ± SEM (*n* = 5 mice group). **(G, H)** Representative flow cytometry profiles gated on gDT-2.CD45.1, OT-2.CD45.1, or SMARTA.CD90.1 CD4^+^ T cells isolated from the spleen showing intracellular levels of IL-17, TNFα, and IFNγ following a brief *in-vitro* stimulation. Green contour plots represent unstimulated controls. **(H)** The graph depicts the proportion of gDT-2.CD45.1, OT-2.CD45.1, and SMARTA.CD90.1 CD4^+^ T cells making IL-17, TNFα, and IFNγ following a brief *in-vitro* stimulation. Bars represent the mean ± SEM (*n* = 5 mice group). **(I–K)** C57BL/6 mice and C57BL/6 mice with memory gDT-2.CD45.1, OT-2.CD45.1, and SMARTA.CD90.1 (generated as described in C) were infected i.v. with 10^6^ CFU of JKD6159–3X^EPI^. Bacterial load in the kidney, nose, lung, and liver on days **(I)** 5 and **(J)** 7 after infection and **(K)** the proportion of colonized mice on day 7 post-challenge was measured (Fisher’s exact test). Symbols represent individual mice, and the bars represent the mean ± SEM (*n* = 10–14 mice per group/time point; two-way ANOVA, Sidak’s multiple comparison). **(L)** The absolute number of gDT.2.CD45.1, OT-2.CD45.1, and SMARTA.CD90.1 CD4^+^ T cells in the spleen, lung, liver, kidney, and kLN on days 5–7 after infection and the proportion on **(M)** day 5 and **(N)** day 7 producing IL-17, TNFα, and IFNγ following a brief *in-vitro* stimulation. Bars represent the mean ± SEM (*n* = 5 mice group, two-way ANOVA, Dunnett’s multiple comparison) *P <0.5, **P <0.01, ***P <0.001, and ****P <0.0001.

The presence of high numbers of monoclonal *S. aureus*-specific circulating effector or memory CD4^+^ T cells alone failed to reduce the severity of an invasive *S. aureus* infection. Although coupling circulating bacteria-specific memory CD4^+^ T cells with boosted numbers of bacterium-reactive Trm at the sites of bacterial replication reduced the severity of *S. aureus* bacteremia, the clinical application of this approach is limited due to challenges associated with establishing Trm in internal organs. Therefore, we investigated whether modifying different aspects of the circulating memory CD4^+^ T-cell pool could improve the efficacy of these T cells against bloodstream *S. aureus* infection. We began by testing whether increasing the breadth of the bacterium-specific circulating memory CD4^+^ T-cell pool could improve protection against invasive *S. aureus* infection. To this end, we generated a recombinant *S. aureus* strain that we engineered to express three known CD4^+^ T-cell epitopes, namely, HSV gD_315–327_, ovalbumin OVA_323–339_, and LCMV gp_61–80_ (**Figure 5A**), and tracked the CD4^+^ T-cell response toward these epitopes using the gDT-2.CD45.1 (gD_315–327_-specific), OT-2.CD45.1 (OVA_323–339_-specific), and SMARTA.CD490.1 (gp_61–80_-specific) CD4^+^ TCR transgenic mice.

We first validated that the three-epitope-expressing *S. aureus* strain, referred to hereafter as JKD6159–3X^EPI^, could trigger gDT-2.CD45.1, SMARTA.CD90.1, and OT-2.CD45.1 CD4^+^ T-cell activation, as measured by an *in-vitro* antigen presentation assay. Splenic DCs and CFSE-labeled gDT-2.CD45.1 or OT-2.CD45.1 or SMARTA.CD90.1 CD4^+^ T cells were cultured together with varying doses of heat-killed JKD6159–3X^EPI^, and the expansion of the transgenic CD4^+^ T cells was assessed 60 h after culture by the dilution of the CFSE dye. All three transgenic CD4^+^ T-cell populations divided in a dose-dependent manner when cultured with JKD6159–3X^EPI^ (**Figure 5B**), and importantly, these transgenic CD4^+^ T cells remained undivided following exposure to the parental JKD6159 strain (data not shown). Next, we generated mice with circulating memory CD4^+^ T cells against all three epitopes and tested their ability to control an invasive *S. aureus* infection. Mice were seeded with 5 × 10^6^ *in-vitro*-activated SMARTA.CD90.1, gDT-2.CD45.1, and OT-2.CD45.1 CD4^+^ T cells and were then rested for 20 days. Despite seeding mice with equal numbers of each transgenic effector CD4^+^ T-cell population, the number of transgenic memory CD4^+^ T cells we recovered from these animals varied, with the number of gDT-2.CD45.1 CD4^+^ T cells routinely exceeding the number of SMARTA and OT-2 CD4^+^ T cells in all tissue compartments profiled (**Figures 5C–F**). The majority of transgenic CD4^+^ memory T cells in the kidney, liver, lung, and nasal tissue were CD62L^−^ Tem, and as expected, we observed minimal Trm development within these sites (**Supplementary Figure 2**). Assessment of the cytokine profile of these memory CD4^+^ T cells following a brief *in-vitro* stimulation with their cognate peptide revealed that all CD4^+^ transgenic T cells were Th1 polarized (**Figures 5G, H**).

To test the protective capacity of this polyclonal memory CD4^+^ T-cell response, naive mice or mice rested for 20 days after seeding with 5 × 10^6^ *in-vitro*-activated SMARTA.CD90.1, gDT-2.CD45.1, and OT-2.CD45.1 CD4^+^ T cells were challenged i.v. with 10^6^ CFU of JKD6159–3X^EPI^. Assessment of the bacterial load in various internal organs after the *S. aureus* challenge revealed that animals with the polyclonal memory CD4^+^ T cells had significantly lower bacterial loads in the kidney on days 5 and 7 post-challenge, respectively (**Figures 5I, J**). This protection was antigen-specific because when we challenged mice with polyclonal memory CD4^+^ T cells with the parental JKD6159 strain, no reduction in bacterial burden was observed in all the profiled tissues (**Supplementary Figure 3A**).

While we observed no statistically significant difference in the *S. aureus* bacterial loads in the liver and nose across both cohorts at both time points, we did observe differences in the colonization frequency in these sites, with 0%–10% of mice with polyclonal bacteria-specific memory CD4^+^ T cells remaining *S. aureus* colonized in these regions on day 7 after challenge, in comparison to 50%–65% colonization in the control cohort (**Figure 5K**). Evaluation of the transgenic CD4^+^ T-cell pools on day 5 and day 7 post-challenge revealed an elevation in the number of all transgenic CD4^+^ T-cell subsets in the kidney, a reduction of all three CD4^+^ T-cell subsets in the liver, while we observed no statistically significant difference in the size of the CD4^+^ T-cell pools in either the spleen or kLN (**Figure 5L**). Despite being largely Th1 polarized prior to systemic *S. aureus* challenge, assessment of the cytokine profile of the CD4^+^ T-cell subsets revealed that on day 5 post-challenge, OT-2.CD45.1 CD4^+^ T cells in the liver and kidney were predominantly Th17 producers, while gDT-2.CD45.1 and SMARTA.CD90.1 CD4^+^ T cells at these sites were Th1 polarized, and this Th1 polarity also dominated in the lung and lymphoid tissue (**Figure 5M**). However, on day 7 post-challenge, all CD4^+^ transgenic T cells in all sites profiled were predominantly Th1 polarized (**Figure 5N**). Thus, polyclonal and polyfunctional circulating memory CD4^+^ T cells can protect against invasive *S. aureus* infection.

In the polyclonal CD4^+^ T-cell transfer experiments described above, we seeded mice with a total of 1.5 × 10^7^ total effector CD4^+^ T cells (5 × 10^6^ of each subset), while in our earlier experiments where we tested the capacity of monoclonal memory CD4^+^ T cells to protect against systemic *S. aureus* infection, a total of 5 × 10^6^ T cells were adoptively transferred into recipient mice. Thus, to test whether the observed protection in mice seeded with a polyclonal memory CD4^+^ T-cell pool was due to the diversity of the T cells and not merely due to the transfer of a larger number of cells, naive mice or mice seeded with 1.5 × 10^7^ *in-vitro*-activated effector gDT-2.CD45.1 CD4^+^ T cells and rested for 20 days were then challenged i.v. with 10^6^ CFU of JKD6159-gD. We observed equal levels of bacteria in the nose, lung, kidney, and liver on days 3, 7, and 14 after infection of naive mice and mice that were recipients of high numbers of gDT-2.CD45.1 CD4^+^ T memory (**Supplementary Figure 3B**). This phenotype was also confirmed when we created mice with high numbers of memory gDT-2.CD45.1 CD4^+^ T cells and challenged them with the JKD6159–3X^EPI^ strain (**Supplementary Figure 3C**). Collectively, these data show that polyclonal but not monoclonal circulating bacterium-specific memory CD4^+^ T-cell response significantly reduced the severity of *S. aureus* bacteremia.

In summary, here, we show that seeding mice with a monoclonal bacterium-specific circulating memory CD4^+^ T-cell population does not protect against *S. aureus* bacteremia. However, the introduction of a polyclonal and polyfunctional circulating memory CD4^+^ T-cell pool significantly reduced the bacterial burden. During the virus infection, T-cell diversity within the memory T-cell pool is known to be advantageous (24). T cells of diverse specificity are believed to result in enhanced killing of virus-infected cells and can protect against and limit the emergence of viral escape variants as these repertoires are more likely to contain cross-reactive and high-avidity T-cell clones. Increasing the breadth of a T-cell response has been shown to improve infection control with candidate vaccines that induced polyclonal T-cell responses conferring enhanced protection against several viral pathogens (25–27). Here, we demonstrate that a polyclonal memory CD4^+^ T-cell pool is also advantageous against systemic bacterial infection. Our findings support the development of a multi-epitope T-cell-based *S. aureus* vaccine, as a strategy to mitigate the severity of *S. aureus* bacteremia. The realization of such a vaccine strategy hinges upon a deeper understanding of highly conserved and immunogenic *S. aureus* T-cell epitopes.

## Data availability statement

The raw data supporting the conclusions of this article will be made available by the authors, without undue reservation.

## Ethics statement

The animal study was approved by The Melbourne University Animal Ethics Committee. The study was conducted in accordance with the local legislation and institutional requirements.

## Author contributions

JB: Conceptualization, Data curation, Formal analysis, Investigation, Writing – review & editing. IM: Conceptualization, Data curation, Methodology, Resources, Writing – review & editing. HZ: Data curation, Writing – review & editing. TS: Resources, Supervision, Writing – review & editing. LW: Conceptualization, Data curation, Formal analysis, Supervision, Writing – original draft, Writing – review & editing.

## Supplementary material

Supplementary Figure 1 Naïve C57BL/6 mice (primary infection) or C57BL/6 mice infected i.v. 28 days earlier (secondary infection) with JKD6159 were infected i.v. with 10^6^ CFU of JKD6159 and at days 3, 5 and 7 post infection the bacterial load in the kidney, nose, lung and liver was measured. Symbols represent individual mice, and the bars represent the mean ± sem.

Supplementary Figure 2 C57BL/6 were seeded with 5 x 10^6^ *in vitro* activated gDT-2.CD45.1, OT-2.CD45.1 and SMARTA.CD90.1 CD4^+^ T cells and were rested for 20 days. The proportion of each transgenic CD4^+^ T cell subset expressing Tcm (CD62L^+^), Tem (CD62L^-^) and Trm (CD69^+^) markers in various tissues were measured. Bars represent the mean ± sem (n = 5).

Supplementary Figure 3
**(A)** C57BL/6 were seeded with 5 x 10^6^ *in vitro* activated gDT-2.CD45.1, OT-2.CD45.1 and SMARTA.CD90.1 CD4^+^ T cells and rested for 20 days were infected i.v. with 10^6^ CFU of JKD6159. Bacterial loads in the kidney, nose, lung, and liver on day 5 post infection were measured. Symbols represent individual mice, and the bars represent the mean ± sem (n = 4–5 mice per group) **(B)** C57BL/6 were seeded with 1.5 x10^7^ *in vitro* activated gDT-2.CD45.1 CD4^+^ T cells and rested for 20 days were infected i.v. with 10^6^ CFU of JKD6159-gD. Bacterial loads in the kidney, nose, lung, and liver at days 3, 7 and 14 post infection were measured. Symbols represent individual mice, and the bars represent the mean ± sem (n = 3–5 mice per group/timepoint) **(C)** C57BL/6 were seeded with 5 x 10^6^ *in vitro* activated gDT-2.CD45.1, OT-2.CD45.1 and SMARTA.CD90.1 CD4^+^ T cells or 1.5 x 10^7^ *in vitro* activated gDT-2.CD45.1, and rested for 20 days were infected i.v. with 10^6^ CFU of JKD6159–3X^EPI^. Bacterial loads in the kidney, nose, lung, and liver on day 5 post infection were measured. Symbols represent individual mice, and the bars represent the mean ± sem (n = 5–14 mice per group, Two-way ANOVA, Sidak’s multiple comparison; data pooled with results presented in ).
