# Supplementary material for: Polyclonal but not monoclonal circulating memory CD4+ T cells attenuate the severity of Staphylococcus aureus bacteremia
Source: Front Immunol. 2024 May 29;15:1417220. doi: 10.3389/fimmu.2024.1417220 (PMC11167101; doi:10.3389/fimmu.2024.1417220)
Supplement: Supplementary Figure 1 — Naïve C57BL/6 mice (primary infection) or C57BL/6 mice infected i.v. 28 days earlier (secondary infection) with JKD6159 were infected i.v. with 106 CFU of JKD6159 and at days 3, 5 and 7 post infection the bacterial load in the kidney, nose, lung and liver was measured. Symbols represent individual mice, and the bars represent the mean ± sem. [file DataSheet_1.docx]

**Supplementary Table 1**

**
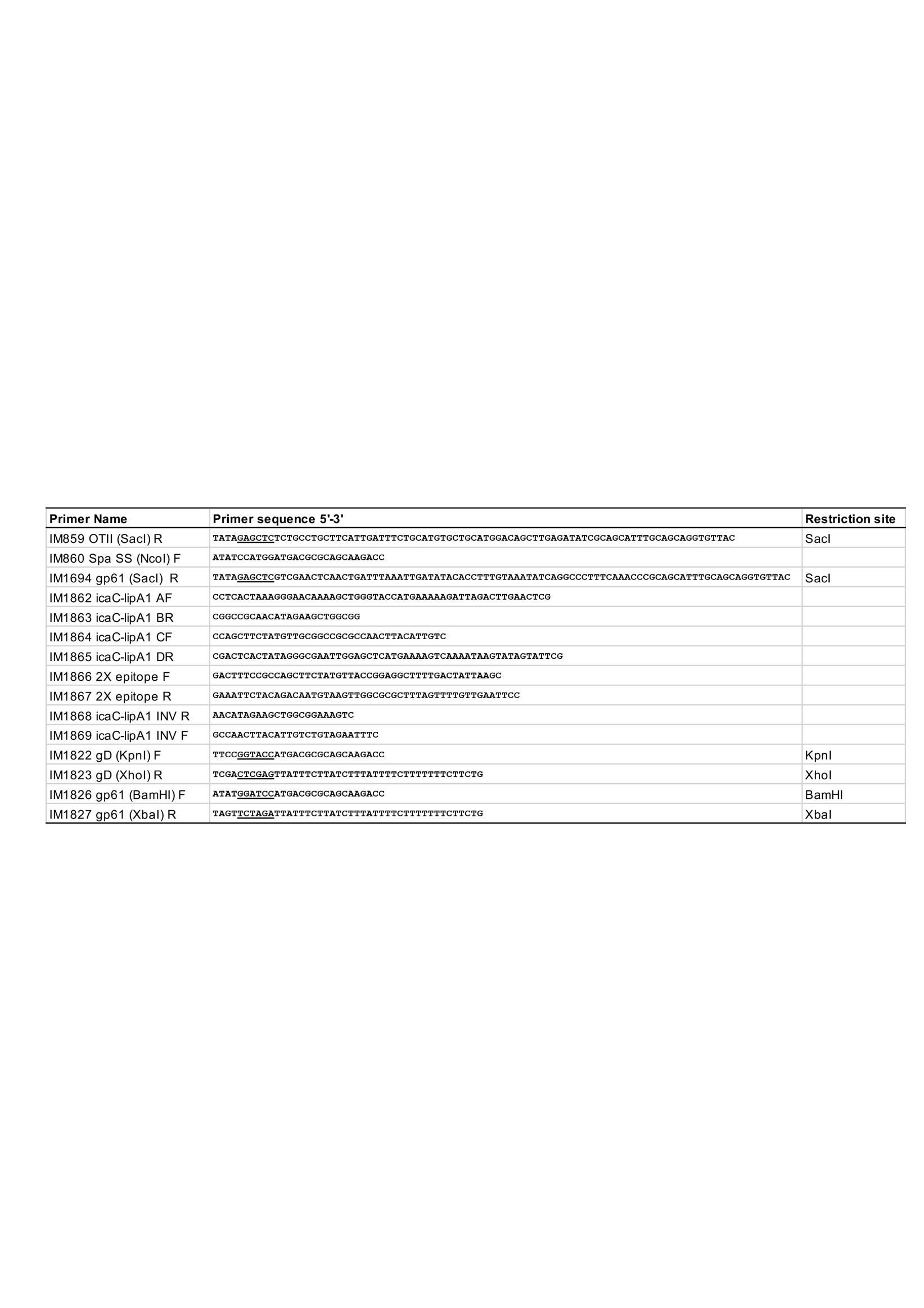
**


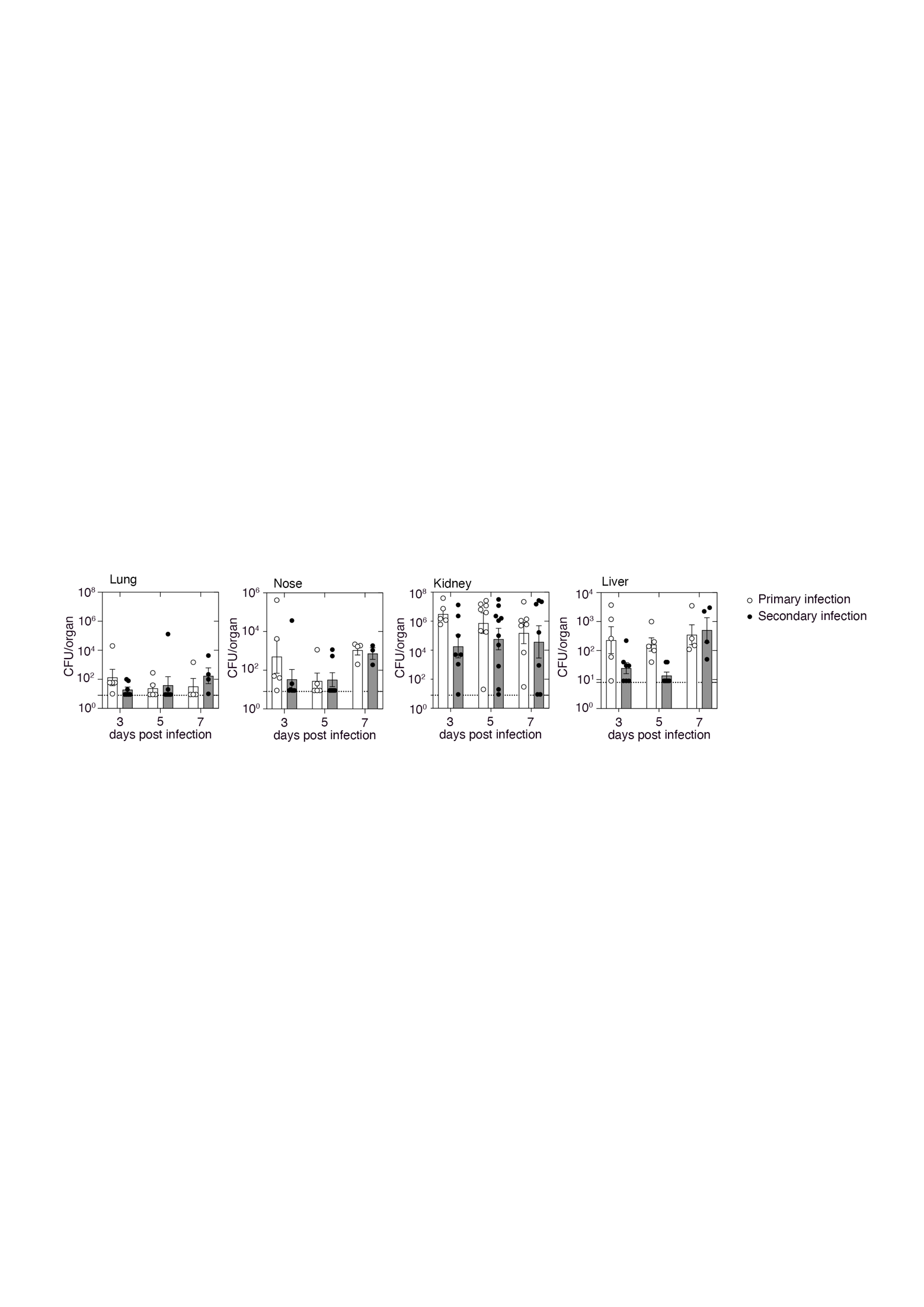


**Supplementary Figure 1**

Naïve C57BL/6 mice (primary infection) or C57BL/6 mice infected i.v. 28 days earlier (secondary infection) with JKD6159 were infected i.v. with 10^6^ CFU of JKD6159 and at days 3, 5 and 7 post infection the bacterial load in the kidney, nose, lung and liver was measured. Symbols represent individual mice, and the bars represent the mean + sem.


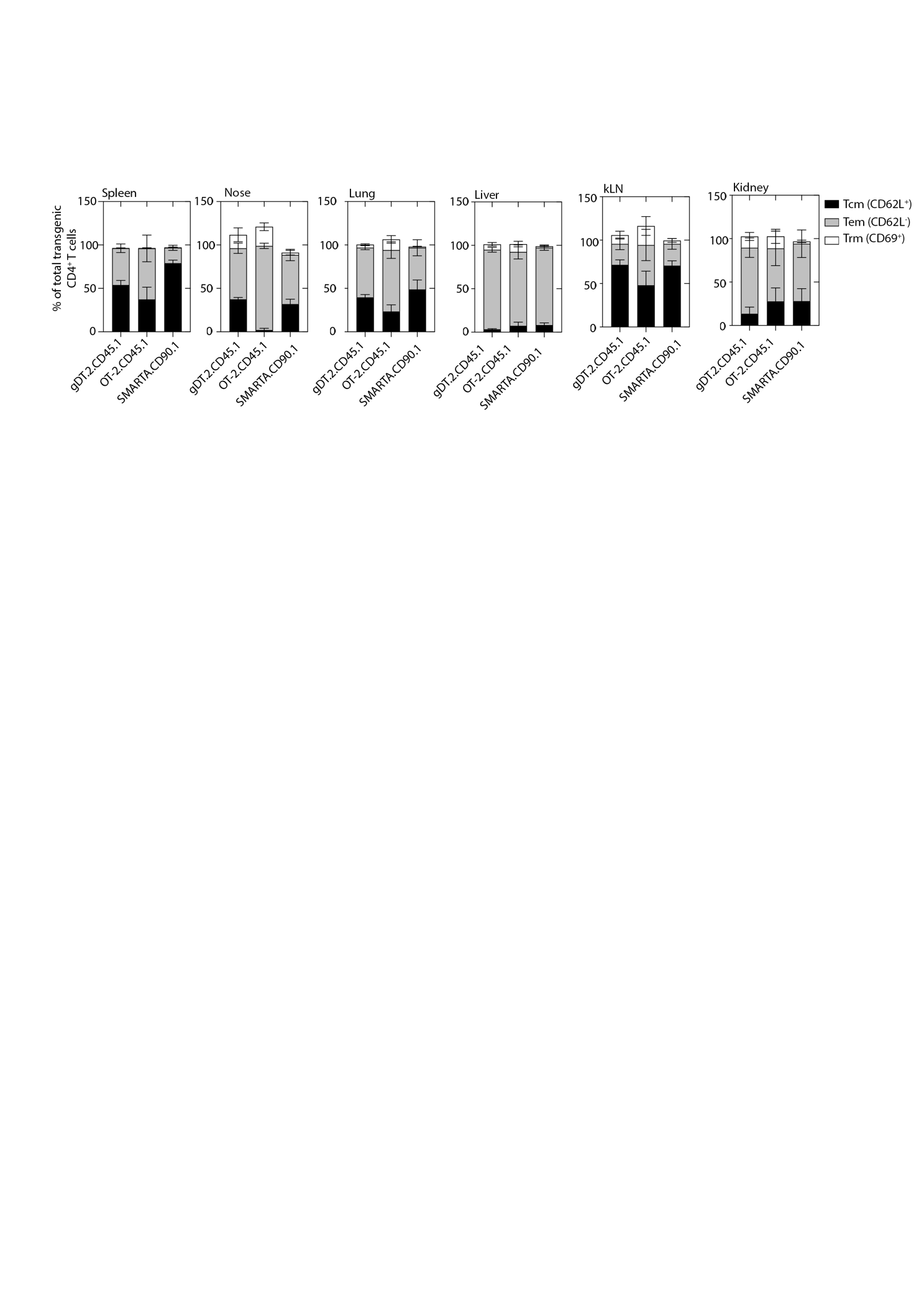


**Supplementary Figure 2**

C57BL/6 were seeded with 5 x 10^6^ *in vitro* activated gDT-2.CD45.1, OT-2.CD45.1 and SMARTA.CD90.1 CD4^+^ T cells and were rested for 20 days. The proportion of each transgenic CD4^+^ T cell subset expressing Tcm (CD62L^+^), Tem (CD62L^-^) and Trm (CD69^+^) markers in various tissues were measured. Bars represent the mean + sem (n = 5).


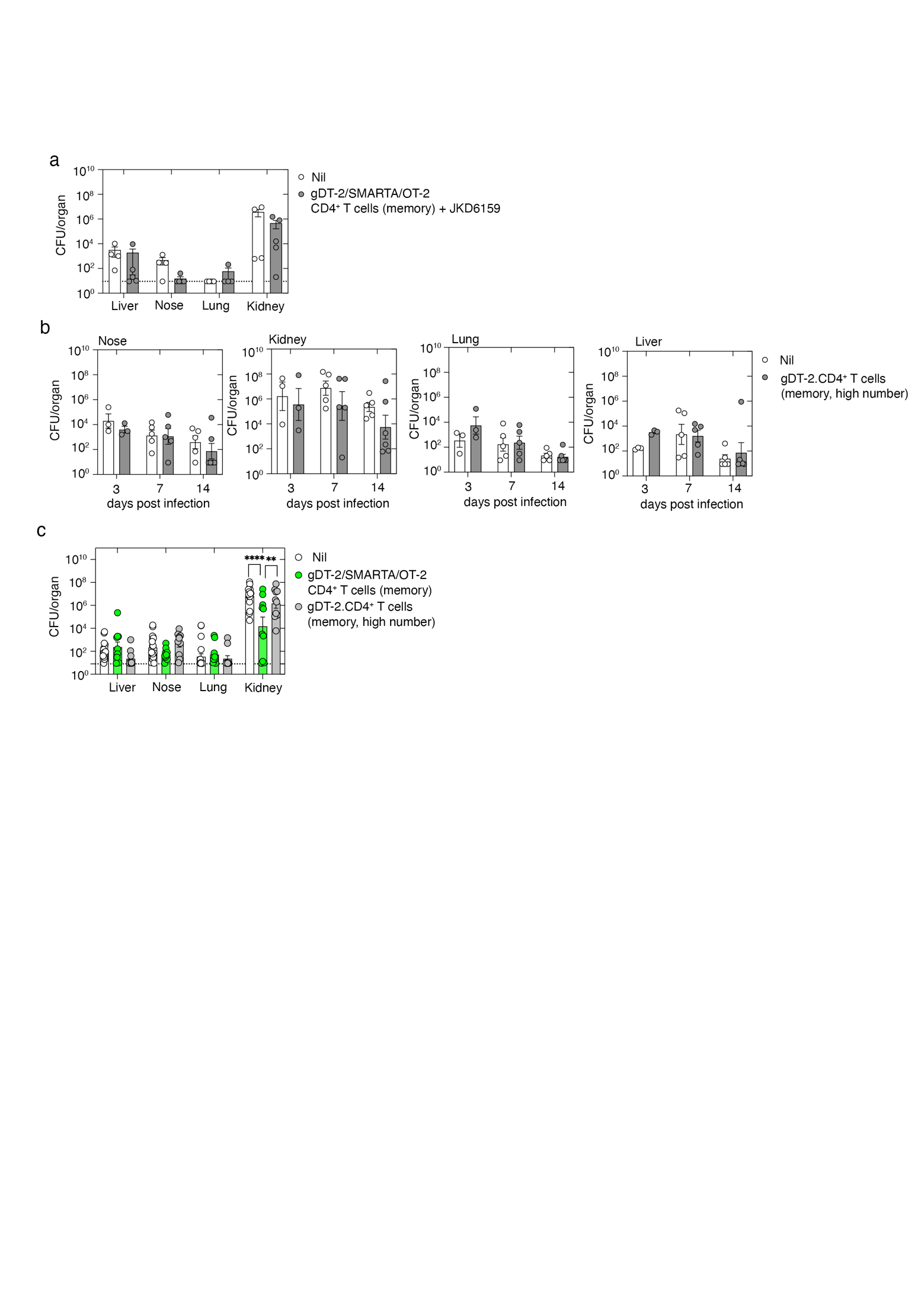


**Supplementary Figure 3**

(a) C57BL/6 were seeded with 5 x 10^6^ *in vitro* activated gDT-2.CD45.1, OT-2.CD45.1 and SMARTA.CD90.1 CD4^+^ T cells and rested for 20 days were infected i.v. with 10^6^ CFU of JKD6159. Bacterial loads in the kidney, nose, lung, and liver on day 5 post infection were measured. Symbols represent individual mice, and the bars represent the mean + sem (n = 4-5 mice per group) (b) C57BL/6 were seeded with 1.5 x10^7^ *in vitro* activated gDT-2.CD45.1 CD4^+^ T cells and rested for 20 days were infected i.v. with 10^6^ CFU of JKD6159-gD. Bacterial loads in the kidney, nose, lung, and liver at days 3, 7 and 14 post infection were measured. Symbols represent individual mice, and the bars represent the mean + sem (n = 3-5 mice per group/timepoint) (c) C57BL/6 were seeded with 5 x 10^6^ *in vitro* activated gDT-2.CD45.1, OT-2.CD45.1 and SMARTA.CD90.1 CD4^+^ T cells or 1.5 x 10^7^ *in vitro* activated gDT-2.CD45.1, and rested for 20 days were infected i.v. with 10^6^ CFU of JKD6159-3X^EPI^. Bacterial loads in the kidney, nose, lung, and liver on day 5 post infection were measured. Symbols represent individual mice, and the bars represent the mean + sem (n = 5-14 mice per group, Two-way ANOVA, Sidak’s multiple comparison; data pooled with results presented in Fig 5i).
